# Three-dimensional simulator: training for beginners in endovascular embolization with liquid agents

**DOI:** 10.1186/s42155-021-00266-y

**Published:** 2021-11-12

**Authors:** Michal Matyjas, Marius Sauerbrey, Sebastian Wyschkon, Maximilian de Bucourt, Michael Scheel

**Affiliations:** 1 Department of Neuroradiology, Charité – Universitätsmedizin Berlin, corporate member of Freie Universität Berlin and Humboldt-Universität zu Berlin, Berlin, Germany; 2 Department of Radiology, Charité – Universitätsmedizin Berlin, corporate member of Freie Universität Berlin and Humboldt-Universität zu Berlin, Berlin, Germany

**Keywords:** Embolization, therapeutic, Simulation, Training, Radiology, interventional, Endovascular procedures

## Abstract

**Background:**

To design a simulator for novices without prior experience in embolization with liquid agents such as n-Butyl cyanoacrylate (n-BCA) and to evaluate the simulator using surveys and post hoc video analysis.

**Materials and methods:**

The simulator was created using computer-aided design software and three-dimensionally printed. Before an embolization, trainees completed questionnaires regarding their level of expertise and self-reported confidence level. The participants were shown an instruction video and each participant performed four embolizations on the simulator. Subsequently, the participants completed surveys on self-reported confidence level and assessed the simulator’s face and content validity.

**Results:**

Five experts and twelve novices trained on the simulator. The experts were radiology residents and fellows with at least 5 years of work experience in interventional radiology. The novices were medical students and radiology residents without any previous experience with embolization. Based on the surveys, the experts assessed the simulator as very useful for embolization training. Performance, e.g. mean duration embolization between experts (mean ± standard deviation = 189 ± 42 s) and novices (mean ± standard deviation = 235 ± 66 s) were significantly different (*p* = .001). The overall simulation of the embolization process, simulated complications, and educational capabilities of the simulator were evaluated positively. In the novice group the self-reported confidence level significantly increased (*p* = .001).

**Conclusion:**

The liquid embolization simulator proposed here is a suitable educational tool for training embolization procedures. It reduces the duration of embolization procedures and improves the confidence level of beginners in embolization.

**Supplementary Information:**

The online version contains supplementary material available at 10.1186/s42155-021-00266-y.

## Background

Simulation training is an educational standard in many different areas of the aviation industry. Aircraft pilots train and need to demonstrate their skills on certified flight simulation training devices (European Parliament, Council of the European Union 2018). In recent years simulation is gaining more and more attention in medical education and training. For example, emergency response units use simulation, to prepare and adapt to changing working environments (Okuda et al. 2008). In radiology, simulators are used to enhance procedural and non-procedural skills (Bartal and Rundback 2018). Simulation-based training is already today a recommended or required part of some residency programs (vascular surgery, interventional cardiology, neurosurgery) (Mandal and Ojha 2020). One important reason for this development is the shortage of training opportunities. Since the advent of computer tomography and magnetic resonance imaging-based angiography, there is a lack of “easy training cases” especially in the field of interventional radiology (IR) (Mirza and Athreya 2018).

IR encompasses a broad spectrum of interventional procedures, including embolization. Embolization with liquid agents has become a widely used treatment for arteriovenous malformations, varicoceles, gastrointestinal bleedings, aneurysms, and pseudoaneurysms (Golzarian et al. 2006). These procedures require advanced haptic skills, knowledge of subsequent steps, and a proper risk assessment. In the field of IR, there is an increasing interest to use simulation-based training, especially for enhancing procedural skills for vascular intervention (Bartal and Rundback 2018). The simulators can be classified into three categories of models: animal models, physical models (e.g. tube models), and virtual reality (VR) simulators (Neequaye et al. 2007). They offer a distinctly unique training experience, prioritizing various characteristics of the simulation.

The animal models offer a few embolization objectives (Grunwald et al. 2006; Fahed et al. 2017). They provide realistic haptic feedback, but come with many ethical concerns, are costly, non-reusable, and preparation of a training environment is complex (Neequaye et al. 2007). The VR-simulators offer various scenarios (e.g. peripheral embolization), can be repeatably used, and objectively assess trainees’ performance (Amin et al. 2019). Their significant disadvantages are relatively high purchasing costs, regular maintaining services, and expensive repairs (Neequaye et al. 2007). Another important disadvantage of most VR-simulators is the absence of liquids, resulting in inadequate depiction of injection-rates and the handling of air bubbles.

In comparison, physical tube models are cheaper and can be used intuitively, without prior training on a training system itself. They allow training of interventional procedures using real instruments and materials with realistic haptic feedback. The main restriction of these models is a limited amount of vascular anatomy and pathology, simulated in one specific model (Neequaye et al. 2007).

To our knowledge, no physical model for embolization procedures with liquid agents is commercially available. Therefore, we wanted to create a model, capable of teaching fundamental procedural steps of the embolization procedure. The model should provide a realistic training environment while being low-cost and feasible for possibility of one-time use models. To achieve a high educational validity of the simulator we followed these steps: 1) defined learning objectives by interviewing IR experts, 2) developed the physical model, 3) evaluated our model in a training with novices and experts in IR.

## Materials and methods

### Learning objectives

The learning objectives were defined and based on interviews with three IR experts (experts having 6, 16 and 24 years of IR experience). The interviews focused on questions regarding specific steps of an embolization procedure, used instruments, characteristics of embolic agents, and accompanying complications. The answers were collected, and precise learning objectives were defined, forming a foundation for the development and the evaluation of the simulator.

### Model construction

The model was sketched, sculpted, and exported as a stereolithography (STL) file using Autodesk Fusion 360 (Autodesk Inc., San Rafael, California) and further modeled in Meshmixer (Autodesk Inc., San Rafael, California). The sculpture was imported to Preform (Formlabs Inc., Somerville, Massachusetts), printed on Formlabs Form 2 (Formlabs Inc., Somerville, Massachusetts), and cured using ultra-violet light with Form cure (Formlabs Inc., Somerville, Massachusetts).

The simulator design purposely depicts abstract targets (chambers) and not specific anatomical regions.

**Fig. 1:**
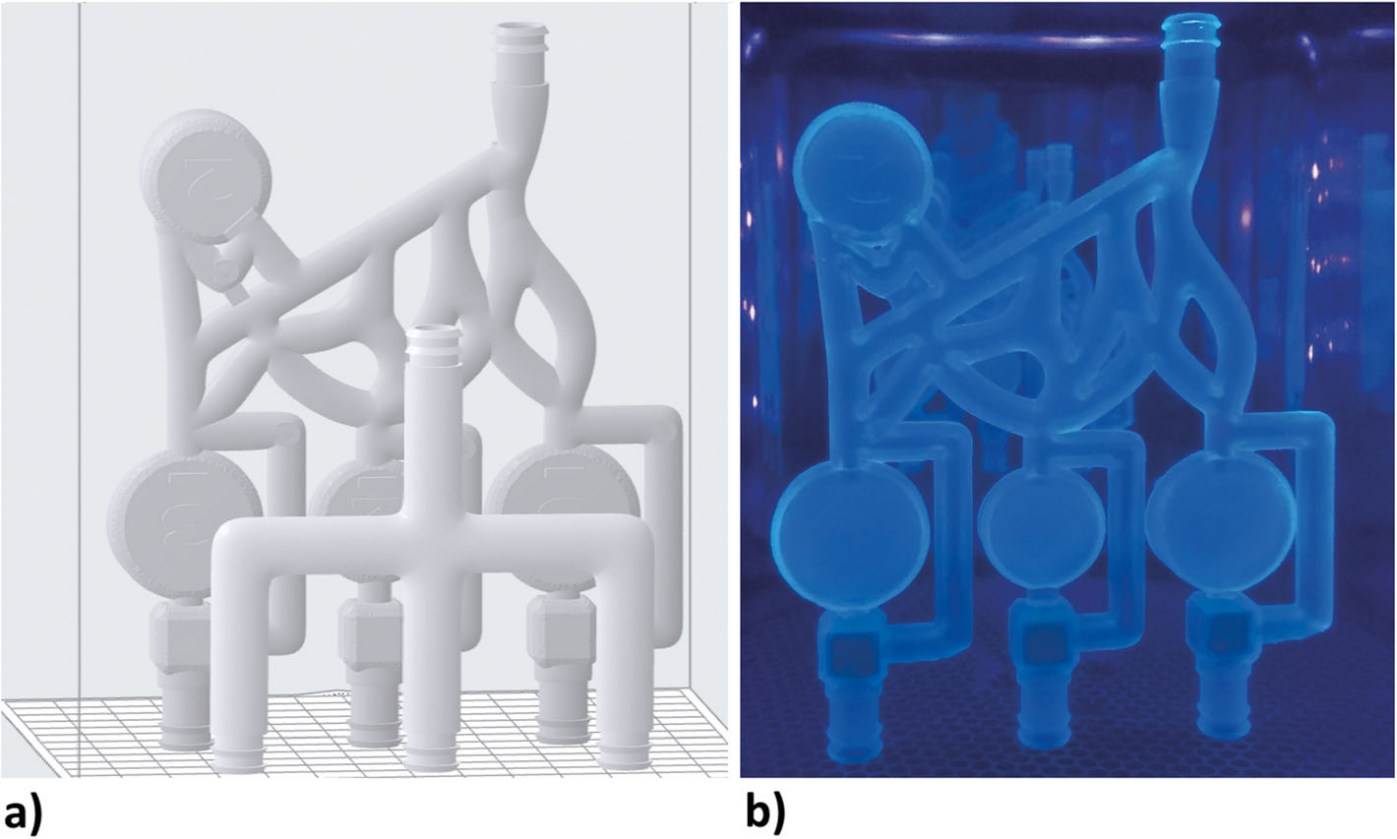
Development stages of the simulator. a) The adapter (anteriorly) and the main body of the simulator (posteriorly) prepared to print in Preform b) The main body cured with ultra-violet light

The model consists of four chambers with adjacent collaterals, interconnected between one another by a network of tubes. The chambers are cylindrical segments having a volume of 2 or 3 ml to sensitize trainees to various quantities of an embolic agent. The design incorporating various chambers should mimic vascular pathologies such as arteriovenous malformations or highly vascularized tumors. At the top are cube-shaped blocks filled with a sponge. These act as a filter, blockading the flow of embolic agent outside of the model. The three outflows are united into a single outflow with an additionally printed adapter. After embolization of all the chambers, only the main component needs to be replaced, while the adapter can be reused (Fig. 1).

The model has a size of 149 × 119 × 21 mm and it takes approximately 7 h and 45 min to print. We used “Clear Resin” from Formlabs as the printing material (Formlabs Inc., Somerville, Massachusetts).

### Model evaluation

**Fig. 2:**
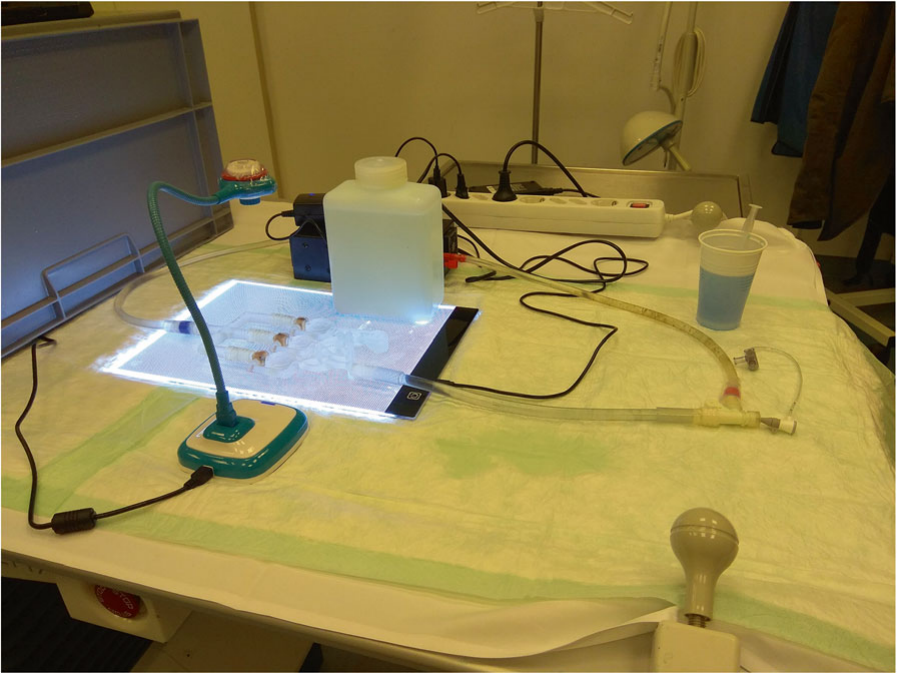
Training environment consisting of the 3d-printed model with connected pump, underlying LED panel and portable camera

**Fig. 3:**
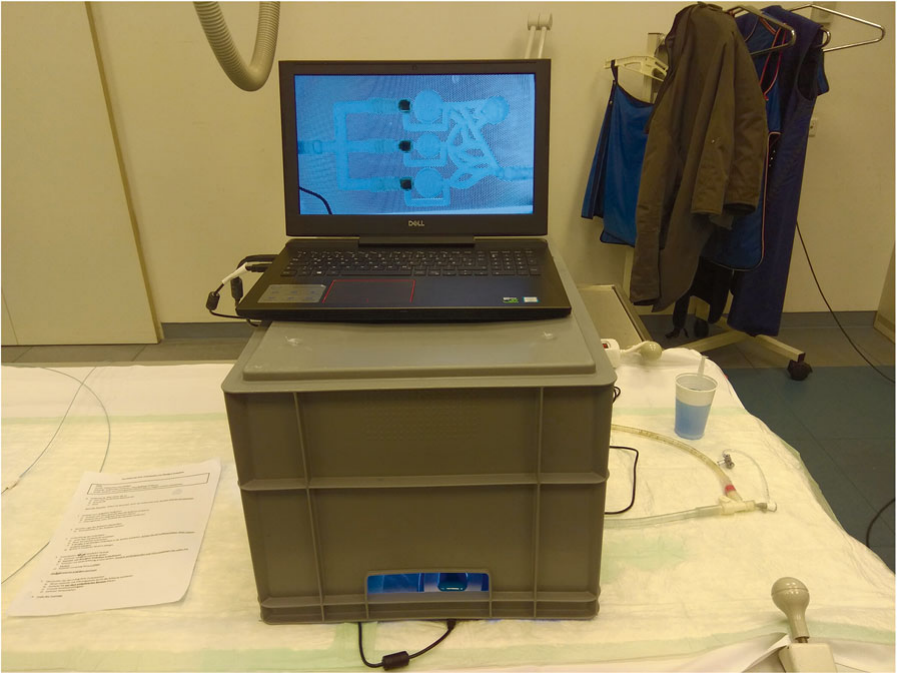
Model, LED panel and camera are hidden inside the plastic box. The laptop, connected to the camera, displaying the model

**Fig. 4:**
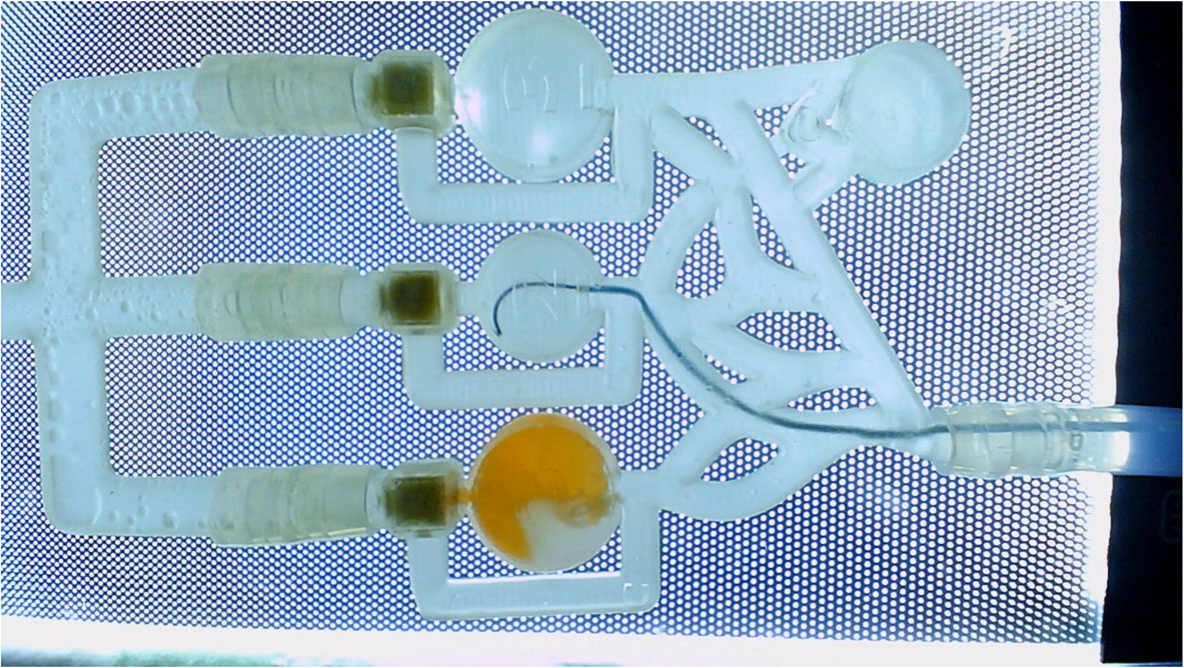
View of the model displayed on the laptop screen during the procedure

We wanted to evaluate our model by two groups: experts and novices. We have defined experts as fellows in radiology with at least 5 years of work experience. Novices were medical students or radiology residents with no prior experience with embolization. Every participant had to perform four embolizations. Participants should identify the given targeted chamber, place the catheter and guide wire in a controlled manner into the predetermined chamber and adjust the necessary amount of embolic agent. The injected amount should be equal to 2 or 3 ml, depending on the targeted chamber. To approach the chamber a 0,035“ angled guide wire (Terumo, Tokyo, Japan) and a 0,038” angiographic catheter (Cordis, California, USA) were used. The simulator was connected to a flow pump (FlowTek 100, United Biologics Inc., Santa Ana, California). Underneath the simulator, a LED panel was placed to increase the visibility of all materials. A camera above the simulator was used to record the training and connected to a laptop for visual feedback (Figs. 2, 3 and 4).

As a low-cost replacement for the embolization agent, we tested different materials. For this purpose, we used the following selection criteria: 1) the material should behave plastically when applied, 2) it should polymerize after application and form a solid body, 3) the material should be non-toxic, 4) it should be widely available. Based on the selection criteria, we identified superglue as an appropriate agent. By comparing viscosities and densities of selected superglues with n-BCA and considering their availability, we decided to use Pattex superglue liquid (Henkel AG & Co, Düsseldorf, Germany) as our primary agent.

The training area of the simulation was divided into “dry” and “wet” areas. In the dry area, participants prepared an embolic agent, where they mixed Pattex Superglue Liquid with red paint pigment for better visibility. In the wet area, the embolic agent was delivered via 3 ml syringes. To substitute a contrast agent, we chose blue food coloring.

To evaluate participants’ effectiveness and measure the time of procedures, we used two cameras: one directly above the simulator and the second one pointed at the participants. The number of occluded chambers, occurence of backflow, number of successfully performed embolizations and the embolization time were assessed with post hoc video analysis.

The occlusion was defined as success when the chamber was closed, with no observable flow of the contrast agent in the control run. Backflow was defined as reflux of the embolic agent, resulting in a closure of the collateral vessel and blockage of contrast agent’s flow. If the chamber was fully occluded and no backflow was observed, the performed embolization was rated as successful. The time of embolization was measured from the moment of the catheter’s introduction through the sheath until retraction of all materials. After the training all participants filled out a questionnaire evaluating the simulator and the overall training.

### Statistical analysis

The number of successfully performed embolizations, closed chambers and backflow occurrences were compared between the novice and the expert group using a chi-quadrat test. The duration of embolizations between the two groups was analyzed using an independent samples Student’s t-test. The changes in proficiency level before and after the training in both groups were compared using a paired samples Student’s t-test. The statistical analysis and figures were performed using R (www.r-project.org).

## Results

### Learning objectives

Based on the interviews, the following learning objectives were defined: 1) handling and navigation of catheter and guidewire, 2) preparation and application of embolic agent, 3) embolization of given target, 4) occlusion’s control with contrast agent and 5) awareness of arising complications, such as catheter’s gluing, insufficient occlusion, backflow, collaterals’ and wrong vessels’ occlusion.

### Model construction

The simulator was constructed as a rather abstract network of tubes with interconnecting chambers. Each chamber acted as embolization target. The model was designed in a 3D modeling software, i.e. Autodesk Fusion 360 (Autodesk Inc., San Rafael, California) and further modeled in Meshmixer (Autodesk Inc., San Rafael, California). The model was then printed on a 3D printer, i.e. Formlabs Form 2 (Formlabs Inc., Somerville, Massachusetts), and cured using ultra-violet light with Form cure (Formlabs Inc., Somerville, Massachusetts).

### Model evaluation

The study involved 17 participants: 12 novices and 5 experts. In the post hoc video analysis, we focused on the overall success rate, the number of occluded chambers, occurrence of backflow, and the duration of an embolization procedure.

**Fig. 5:**
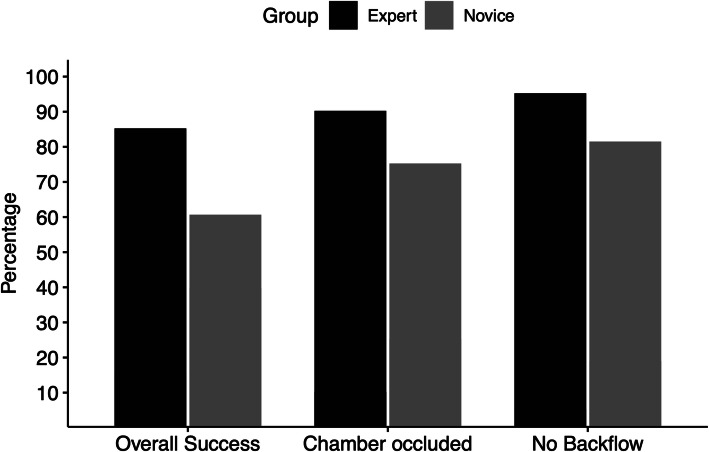
Rates (in %) for overall success, complete chamber occlusion and prevention of backflows

The overall success rate for the embolization procedure was 85% in the expert group and 60% in the novice group (*p* = .048). The experts successfully occluded 18 (90%) chambers and the novices 36 (75%) (*p* = .163). No backflow of the embolic agent occurred in 19 (95%) embolizations in the expert group and 39 (81%) in the novice group (*p* = .145) (Fig. 5).

Additionally, we measured the procedure duration, i.e. until the catheter was fully retracted. The duration exceeded 10 min in 3 cases (all in the novice group). These trials were excluded from the following analysis.

**Fig. 6:**
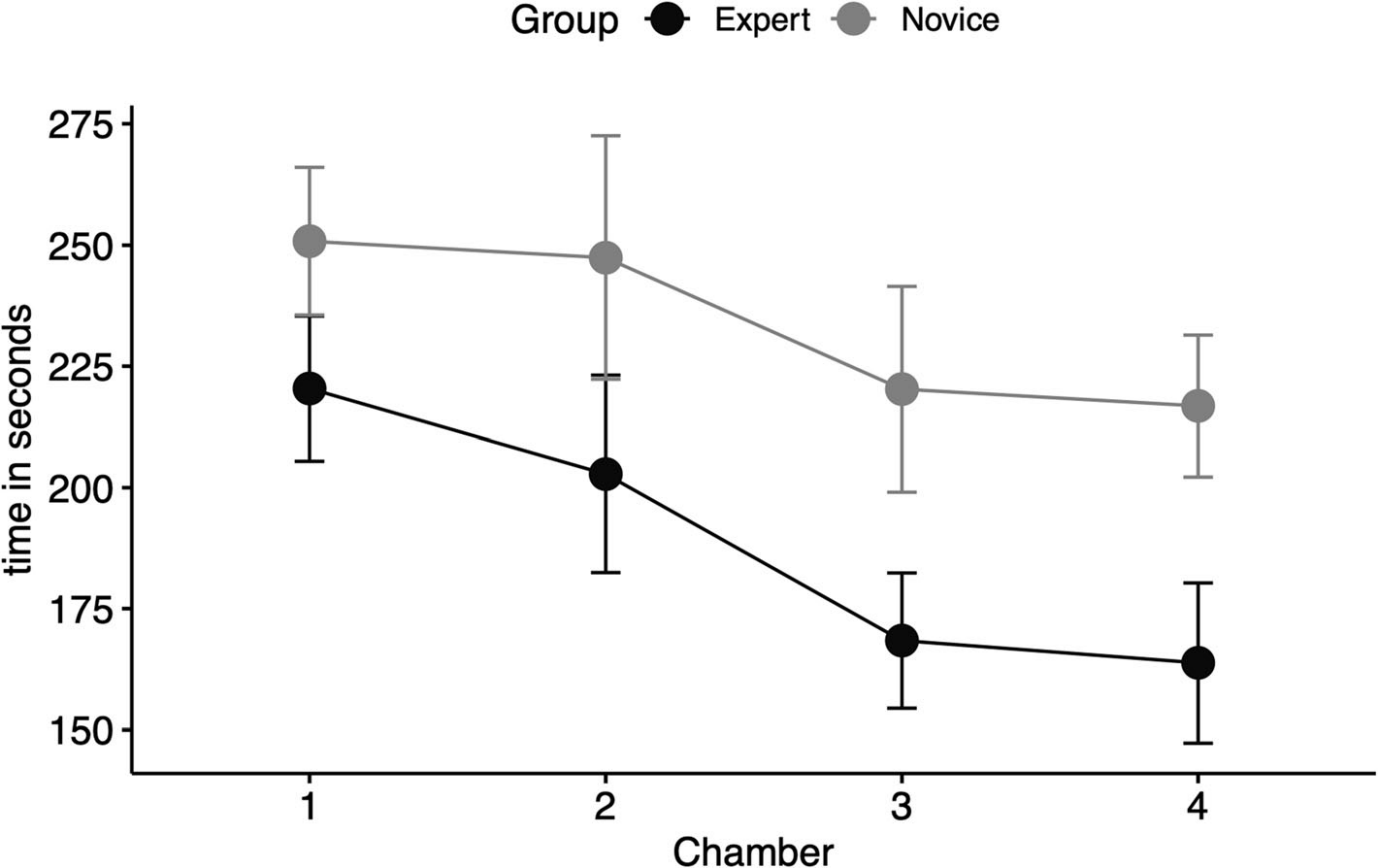
Comparison of procedure times in seconds (mean ± SE (standard error)) between experts and beginners

In the expert group the mean embolization duration was 189 ± 42 s (mean ± standard deviation (SD)) and 235 ± 66 (mean ± SD) seconds in the novice group (*p* = .001). The embolization duration significantly decreased during the training in both groups (Fig. 6).

To evaluate the realism of our simulator, we asked experts and beginners about the trained tasks and complications (Tables 1 and 2). To assess the simulator’s training potential, we asked the experts, if our model could be used as a training tool (Table 3).

**Table 1:** Summarized answers of the participants to the questionnaire regarding the simulator (*n* = 17)

The model simulates the following tasks:	Strongly agree	Agree	Neither	Disagree	Strongly disagree
Navigation of the catheter and guidewire	8 (47)	9 (53)	0	0	0
Preparation of the embolic agent	9 (53)	7 (41)	1 (6)	0	0
Application of the embolic agent using the sandwich technique	11 (65)	5 (29)	1 (6)	0	0
Application of the contrast agent	10 (59)	7 (41)	0	0	0
Occlusion of the targeted vessel	6 (35)	11 (65)	0	0	0
The entire embolization procedure	5 (29)	9 (53)	3 (18)	0	0

**Table 2:** Summarized answers of the participants to the questionnaire regarding the complications (*n* = 17)

The model simulates the following complications:	Strongly agree	Agree	Neither	Disagree	Strongly disagree
Wrong vessel occlusion	10 (59)	6 (35)	1 (6)	0	0
Collateral vessel occlusion	11 (65)	6 (35)	0	0	0
Backflow of the embolic agent	12 (71)	5 (29)	0	0	0
Insufficient occlusion of the targeted vessel	8 (47)	9 (53)	0	0	0
Catheter’s gluing	8 (47)	7 (41)	1 (6)	1 (6)	0
The identification and prevention of general complications	4 (24)	12 (71)	1 (6)	0	0

**Table 3:** Summarized answers of the experts to the questionnaire regarding educational validity (*n* = 5)

The model	Strongly agree	Agree	Neither	Disagree	Strongly disagree
Trains hand-eye coordination	4 (80)	1 (20)	0	0	0
Teaches procedural steps of embolization	4 (80)	1 (20)	0	0	0
Is well suited for the training of beginners in transcatheter embolization	2 (40)	3 (60)	0	0	0
Would be incorporated into a hospital’s residency program	2 (40)	2 (40)	1 (20)	0	0

Identical questions regarding the knowledge and proficiency level were evaluated before and after the training. The novices showed a significant increase in the self-reported knowledge and proficiency level (*p* < .001) (Table 4), while this was not the case for the expert group.

**Table 4:** Differences in self-reported assessment of the novices before and after the training (*n* = 12)

Outcome	pre-training	post-training	***P***-value
Overall, I understand the embolization procedure with a liquid embolic agent	4,1	1,6	< .001
I know all the steps of the embolization procedure	4,5	2,1	< .001
I can reliably handle liquid embolization agent	4,8	2,5	< .001
I know the instruments needed for embolization procedures	4,3	2,0	< .001
I can independently perform embolization procedures	4,8	3,8	< .001
Total	4,5	2,4	< .001

## Discussion

The aim of this study was the construction and evaluation of a physical embolization simulator. We defined learning objectives by interviewing IR experts. Subsequently a 3d-printed simulator was created and evaluated through surveys and post hoc video analysis.

Differences in procedure success rates and procedure times between beginners and experts were demonstrated as a proof of the construct validity of the simulator. A reduction of procedure times could also be demonstrated reflecting the training effect. The educational capabilities of the simulator were evaluated ubiquitously positive by beginners and experts.

The simulator is intended to be the first practical experience for endovascular trainees in the transcatheter embolization with liquid embolic agents. The simulator shall serve as a teaching platform for learning procedural steps, handling embolic agents, familiarizing with the instruments, and highlighting possible complications. The educational validity of the simulators is a measurement of how reliant a simulator can convey knowledge and skills. In our study, we tested construct, face, and content validities. Construct validity identifies the level of expertise between the training groups (Bartal and Rundback 2018). Using video recordings and measuring the outcomes, we have observed differences between the experts and the novices. The experts occluded more chambers and made fewer mistakes (Fig. 4). We have observed significant differences between the experts and the novices in the duration of embolization, as well as the reduction of time required for occlusion (Fig. 5).

The face and content validities are basic parameters demonstrating the simulator’s representation of the trained tasks and its’ teaching potential. (Bartal and Rundback 2018). In the surveys, most of the trainees evaluated our model and trained complications positively. The only negative opinion was regarding the authenticity of the catheter’s gluing (Table 2). The simulator demonstrated a high educational value, based on the experts’ surveys. The experts positively evaluated the teaching potential of the simulator and would incorporate our simulator into a hospital’s residency program (Table 3). The self-reported change in the confidence level of the trainees is a measure of the subjective increase in their competence. We have observed significant improvements in the novice group on self-reported skill and knowledge level about every asked item (*p* < .001). The improvement in the management and understanding of the embolization demonstrates the training capability of the simulator. The smallest increase was observed in the confidence level of the independently performed procedure (Table 4). It is consistent with our intention to design the simulator intended to provide first experiences with embolization and not professional independence.

We believe simulation training should be an integral part of the IR residency curriculum. The proposed simulator could help inexperienced residents and provide a teaching platform for their first embolization experiences. Many institutions have already recognized the benefits of simulation-based training. The Cardiovascular and Interventional Radiological Society of Europe (CIRSE) in its’ current, second edition curricula from the year 2017, supports practice on simulators as a valid method of formal teaching and independent self-directed learning, contributing to growing professionalism (Curriculum/Syllabus 2019). The Royal College of Radiologists in the 2021 curriculum supports a simulation “as a useful tool to supplement training in clinical situations” (The Royal College of Radiologists 2020). Basic vascular intervention and angiography were mentioned as the essential procedures requiring simulation-based training in radiology (Nayahangan et al. 2018).

Endovascular simulators can be divided into animal, physical, and VR simulators (Neequaye et al. 2007). They differ from each other on fidelity levels, reusability, ethical issues, purchase, and maintenance costs. In animal models, anesthetized animals undergo embolization procedures to train and evaluate, established, and new embolization techniques (Naggara et al. 2010; Wilkins et al. 2017; Izaaryene et al. 2016). Those models provide excellent haptic feedback. Animal models however impose ethical and legal issues, are non-reusable, and offer a narrow range of possible simulations. They are problematic in transportation and storage. Preconditioned vascular pathologies, sedation of animals, monitoring of vital signs, and postoperative care generate additional costs (Neequaye et al. 2007; Berry et al. 2008). The physical simulators are devices typically replicating anatomical regions and are limited to the teaching of distinct technical procedures. They serve in the training of ultrasound-guided needle procedures, catheters’ and guidewires’ navigation, and stents’ placement (Berry et al. 2016; Mendiratta-Lala et al. 2010). The physical simulators are low-cost, easily transportable, and do not require an angiographic suite. The lack of multiple training scenarios and non-standardized evaluation are the disadvantages (Neequaye et al. 2007). The VR simulators use computer models of human vasculature, that can be manipulated using simulated or actual medical devices. They offer standardized training scenarios, improving procedural skills i.e. vascular trauma management, uterine and prostatic artery embolization (Mandal and Ojha 2020; Lonn et al. 2012). Those simulators are reusable, provide feedback, measure procedure and fluoroscopy times. However, the high-end equipment, standardized to mimic clinical cases increases the production, purchase, and service costs of the VR simulators (Neequaye et al. 2007; Berry et al. 2008).

The liquid embolization simulator seems to be advantageous when it comes to precisely gain experience in embolization with liquid agents. We intentionally aimed for a low-cost model. The amount of resin needed to print a single simulator, results in production costs of 12 $ per unit. We estimate the per procedure cost for IR materials at 55 $. This includes protective gear, new catheter for every single embolization, materials essential for embolization in sandwich technique and for model production. The overall costs including previously mentioned materials and remaining equipment, such as camera, introducer sheath and guide wire were estimated at 285 $.

Cost reduction, ease of implementation into clinical routine, small size, portability, and absence of ethical issues are advantages in comparison to animal and VR counterparts. The simplified anatomy, use of the real instruments, absence of costly liquid embolics (n-BCA, Onyx) and simulation of basic physiology (blood flow) create an adequate environment for embolization training with liquid agents, especially for inexperienced users.

Certain limitations can be attributed to our simulator and embolization training. Overall, we evaluated only a small group and we did not assess if the skills learned by our participants transfer to the procedures performed in real medical interventions on patients. To show a significant learning curve more training sessions with larger groups and follow-ups would be required.

In our opinion, our simulator enables effective embolization training in a friendly learning environment. The simulator provides the first hands-on experience of the embolization with the liquid agents. It offers inexpensive training opportunities for endovascular trainees and can serve as an additional element of the endovascular training.

## Supplementary Information

**Additional file 1.**

## Data Availability

The datasets used and analyzed during the current study are available from the corresponding author on reasonable request.

## Abbreviations

n-BCA: n-Butyl cyanoacrylate
IR: Interventional radiology
VR: Virtual reality
STL: Stereolithography
SD: Standard deviation
CIRSE: Cardiovascular and Interventional Radiological Society of Europe
SE: Standard error

## References

[CR1] Amin A, Salsamendi J, Sullivan T (2019). High-Fidelity endovascular simulation. Tech Vasc Interv Radiol.

[CR2] Bartal G, Rundback JH, Keefe NA, Haskal ZJ, Park AW, Angle JF (2018). Simulation training in interventional radiology. IR Playb Compr Introd Interv Radiol.

[CR3] Berry E, Marsden A, Dalgarno KW, Kessel D, Scott DJA (2016). Flexible tubular replicas of abdominal aortic aneurysms: Proc Inst Mech Eng [H].

[CR4] Berry M, Hellström M, Göthlin J, Reznick R, Lönn L (2008). Endovascular training with animals versus virtual reality systems: an economic analysis. J Vasc Interv Radiol.

[CR5] Curriculum/Syllabus [Internet]. CIRSE. [cited 2019 Mar 10]. Available from: https://www.cirse.org/education/european-curricula/

[CR6] European Parliament, Council of the European Union. Regulation (EU) 2018/1139 of the European Parliament and of the Council of 4 July 2018 on common rules in the field of civil aviation and establishing a European Union Aviation Safety Agency, and amending Regulations (EC) No 2111/2005, (EC) No 1008/2008, (EU) No 996/2010, (EU) No 376/2014 and Directives 2014/30/EU and 2014/53/EU of the European Parliament and of the Council, and repealing Regulations (EC) No 552/2004 and (EC) No 216/2008 of the European Parliament and of the Council and Council Regulation (EEC) No 3922/91 (Text with EEA relevance). OJ L, 32018R1139 2018. Available from: http://data.europa.eu/eli/reg/2018/1139/oj/eng

[CR7] Fahed R, Gentric JC, Salazkin I, Gevry G, Raymond J, Darsaut TE (2017). Flow diversion of bifurcation aneurysms is more effective when the jailed branch is occluded: an experimental study in a novel canine model. J Neurointerventional Surg.

[CR8] Golzarian J, Siskin GP, Sharafuddin M, Mimura H, Coldwell DM, Golzarian J, Sun S, Sharafuddin MJ (2006). Embolization Tools. Vasc Embolotherapy Compr Approach Vol 1 Gen Princ Chest Abdomen Gt Vessels.

[CR9] Grunwald IQ, Romeike B, Eymann R, Roth C, Struffert T, Reith W (2006). An experimental aneurysm model: a training model for neurointerventionalists. Interv Neuroradiol J Peritherapeutic Neuroradiol Surg Proced Relat Neurosci.

[CR10] Izaaryene J, Saeed Kilani M, Rolland P-H, Gaubert J-Y, Jacquier A, Bartoli J-M, Vidal V (2016). Preclinical study on an animal model of a new non-adhesive cyanoacrylate (Purefill®) for arterial embolization. Diagn Interv Imaging.

[CR11] Lonn L, Edmond JJ, Marco J, Kearney PP, Gallagher AG (2012). Virtual reality simulation training in a high-fidelity procedure suite: operator appraisal. J Vasc Interv Radiol JVIR.

[CR12] Mandal I, Ojha U (2020) Training in Interventional Radiology: A Simulation-Based Approach. J Med Educ Curric Dev 7 [cited 2020 Oct 31]. Available from: https://www.ncbi.nlm.nih.gov/pmc/articles/PMC7155237/10.1177/2382120520912744 PMC7155237 32313840

[CR13] Mendiratta-Lala M, Williams T, de Quadros N, Bonnett J, Mendiratta V (2010). The use of a simulation center to improve resident proficiency in performing ultrasound-guided procedures. Acad Radiol.

[CR14] Mirza S, Athreya S (2018). Review of simulation training in interventional radiology. Acad Radiol.

[CR15] Naggara O, Darsaut TE, Salazkin I, Soulez G, Guilbert F, Roy D, Weill A, Gevry G, Raymond J (2010). A new canine carotid artery bifurcation aneurysm model for the evaluation of neurovascular devices. Am J Neuroradiol.

[CR16] Nayahangan LJ, Nielsen KR, Albrecht-Beste E, Bachmann Nielsen M, Paltved C, Lindorff-Larsen KG, Nielsen BU, Konge L (2018). Determining procedures for simulation-based training in radiology: a nationwide needs assessment. Eur Radiol.

[CR17] Neequaye SK, Aggarwal R, Van Herzeele I, Darzi A, Cheshire NJ (2007). Endovascular skills training and assessment. J Vasc Surg.

[CR18] Okuda Y, Bond W, Bonfante G, McLaughlin S, Spillane L, Wang E, Vozenilek J, Gordon JA (2008). National Growth in simulation training within emergency medicine residency programs, 2003–2008. Acad Emerg Med.

[CR19] The Royal College of Radiologists. Clinical radiology curriculum. [cited 2020 Nov 2]. Available from: https://www.rcr.ac.uk/clinical-radiology/specialty-training/curriculum/clinical-radiology-curriculum

[CR20] Wilkins LR, Stone JR, Mata J, Hawrylack A, Kubicka E, Brautigan DL (2017). The use of the woodchuck as an animal model for evaluation of Transarterial embolization. J Vasc Interv Radiol.

