# Supplementary material for: Three-dimensional simulator: training for beginners in endovascular embolization with liquid agents
Source: CVIR Endovasc. 2021 Nov 12;4:78. doi: 10.1186/s42155-021-00266-y (PMC8589927; doi:10.1186/s42155-021-00266-y)
Supplement: Supplementary file 1 — Additional file 1. [file 42155_2021_266_MOESM1_ESM.docx]

**Appendix:**

**Interview questions to the IR-Experts:**

What are the fundamentals and basics of embolization with liquid agents?

What are the steps of successful embolization?

What materials are usually used for the occlusion?

What are the necessary skills to perform embolization with liquid agents?

What are the possible complications arising during embolization with liquid agents?

How could the participants demonstrate desired skills?

How could we test the learned abilities of the participants?

What requirements would the model have to fulfill to provide essential training for the novices?
